# Respiratory tract mortality in cement workers: a proportionate mortality study

**DOI:** 10.1186/1471-2466-12-30

**Published:** 2012-06-27

**Authors:** George Rachiotis, Spyros Drivas, Konstantinos Kostikas, Vasilios Makropoulos, Christos Hadjichristodoulou

**Affiliations:** 1 Department of Hygiene and Epidemiology, Medical Faculty, University of Thessaly, Larissa, Greece; 2 Greek Institute for Occupational Safety and Health (ELINYAE), Athens, Greece; 3 Department of Occupational and Industrial Medicine, National School Public Health, Athens, Greece

## Abstract

**Background:**

The evidence regarding the association between lung cancer and occupational exposure to cement is controversial. This study investigated causes of deaths from cancer of respiratory tract among cement workers.

**Methods:**

The deaths of the Greek Cement Workers Compensation Scheme were analyzed covering the period 1969-1998. All respiratory, lung, laryngeal and urinary bladder cancer proportionate mortality were calculated for cement production, maintenance, and office workers in the cement industry. Mortality from urinary bladder cancer was used as an indirect indicator of the confounding effect of smoking.

**Results:**

Mortality from all respiratory cancer was significantly increased in cement production workers (PMR = 1.91; 95% CI 1.54 to 2.33). The proportionate mortality from lung cancer was significantly elevated (PMR = 2.05; 95% CI 1.65 to 2.52). A statistically significant increase in proportionate mortality due to respiratory (PMR = 1.7; 95% CI 1.2 to 2.34). and lung cancer (PMR = 1.67;95% CI = 1.15-2.34) among maintenance workers has been observed. The PMR among the three groups of workers (production, maintenance, office) did differ significantly for lung cancer (p = 0.001), while the PMR for urinary bladder cancer found to be similar among the three groups of cement workers.

**Conclusion:**

Cement production, and maintenance workers presented increased lung and respiratory cancer proportionate mortality, and this finding probably cannot be explained by the confounding effect of smoking alone. Further research including use of prospective cohort studies is needed in order to establish a causal association between occupational exposure to cement and risk of lung cancer.

## Background

The production of cement is related to multistage processes that include quarrying, crushing, raw milling, blending, and production of clinker, milling and, last, packing. Cement workers are exposed to a variety of occupational hazards, one of the most important being dust exposure. The main routes of workers’ exposure to cement dust are: dermal, and inhalation. There is considerable evidence that cement workers are at increased risk of respiratory symptoms [1,2]. Most often obstructive airway disease has been the issue of investigation [3]. There is contradictory evidence of an association between exposure to cement dust and lung function impairment [3,4]. This is also the case for the association between exposure to cement dust and respiratory tract malignancies, in particular lung and larynx cancer [5-10]. Nevertheless, it is notable that almost all studies evaluating the association between cement dust and respiratory cancer have included subjects from one or two cement factories, resulting in a low generalizability of the observed results. Greece is a significant cement producing country. In 2007 the annual production of cement in Greece was estimated at 15 million tons [11]. The aim of our study was to study the respiratory cancer mortality among cement workers by the use of the records of the Cement Workers Compensation Scheme in Greece.

## Methods

The records of the Greek Cement Workers Compensation Scheme were examined covering the period 1969-1998. This Scheme was established in 1937. All workers in the cement industry (production workers, maintenance workers and clerical workers) were registered with this Scheme. The total population at risk was not available and consequently, the only feasible choice of study design was the record of the deaths and the calculation of the proportionate mortality. Inclusion criteria were: male sex, known cause of death and minimum duration of employment ≥1 year. The information collected included sex, age at death, starting and ending dates of all employments. Information on causes of death was obtained by the use of death certificates. Causes of death were classified according to the International Classification of Diseases, 9^th^ revision (ICD-9). The codes used were:

Lung cancer (162), larynx (161), and all respiratory cancers (161-163). Urinary bladder cancer data (ICD-9 code 188) were used as a surrogate for the control of the confounding effect related to smoking. Moreover, proportionate mortality from ischemic heart disease, cerebrovascular diseases, all cancer and pneumonia was calculated. The expected number of deaths was calculated based on 5-year age groups and calendar periods. Cause-specific mortality rates for the Greek population were obtained by the Greek National Statistics Service. Proportionate Mortality Ratio (PMR) was calculated for all respiratory, lung, larynx, and bladder cancer mortality. In order to compare qualitatively the PMR between different categories of workers, three categories of exposure to cement dust have been considered. Cement production workers have been considered as the "high exposure group", maintenance workers were considered as the "medium exposure group", and office workers have been considered as the "low exposure group". The lung cancer proportionate mortality of these groups has been compared to that of the general population. In addition, all respiratory, and lung cancer mortality were compared among the three categories of exposure. The protocol of the study has been approved by the scientific committee of the postgraduate program: public and environmental hygiene of the University of Thessaly.

### Statistical analysis

PMRs were calculated by dividing the observed number of deaths by the expected number of deaths. Statistical significance of the results was assessed by the calculation of 95% Confidence Intervals (CI). A Poisson distribution for the observed deaths was assumed. Proportionate mortality among the three categories of exposure was compared with the use of natural logarithms for Analysis of Variance (ANOVA). The level of statistical significance was set at 0.05. Statistical analysis was conducted by the use of Excel, Open Epi and SPSS software.

## Results

Data from 1157 death certificates fulfilled the inclusion criteria. We identified 632 deaths of cement production workers, 336 deaths of maintenance blue collar personnel, and 189 deaths of office workers. Table 1 depicts the socio-demographic characteristics of the cement workers under study. The mean of the age at death for production, maintenance, and office workers was 68, 68.4 and 72 years, respectively. Table 2 illustrates the distribution of the deaths among the three categories of cement workers. It is of interest that for all categories of cement workers the diseases of circulatory system were the prevalent cause of death. Table 3 shows the proportionate mortality of cement workers. Mortality from respiratory cancer was significantly increased in cement production workers In particular, 91 deaths related to cancer of respiratory tract were recorded (85 deaths from lung and 6 from larynx cancer); PMR = 1.91; 95% CI 1.54 to 2.33). The proportionate mortality from lung cancer was significantly elevated (PMR = 2.05; 95% CI 1.65 to 2.52). However, the mortality due to larynx cancer was not significantly increased (PMR = 1.32; 95% CI 0.53 to 2.74). Stratification of PMR by age group has shown that 37 deaths from respiratory cancer have been recorded at the age group </= 64 years (PMR = 1.55;95% CI = 1.1-2.12) and 54 deaths from respiratory cancer have been found at the age group <64 years (PMR = 2.25;95% C.I = 1.7-2.9).

**Table 1:** Demographic characteristics of the study population

**Cement production workers**	
Number of deaths	632
Age at death(mean, range)	68 (27-102)
Starting date of employment(range)	1912-1989
Ending date of employment (range)	1949-1998
**Blue-collar maintenance workers**	
Number of deaths	336
Age at death(mean, range)	68.4 (31-110)
Starting date of employment(range)	1919-1995
Ending date of employment (range)	1948-1998
**Office workers**	
Number of deaths	189
Age at death(mean, range)	72 (32-100)
Starting date of employment(range)	1910-1984
Ending date of employment (range)	1945-1998

**Table 2:** Distribution of the deaths among cement workers in Greece (1969-1998)

**Causes of death**	**N/Total (%)**
**Production cement workers**	
Infectious diseases (0-139)	13/632 (2,1)
Diseases of genitourinary system (580-629)	3/632 (0,5)
Accidents and violence (800-999)	30/632 (4,7)
Diseases of the musculoskeletal system and connective tissue (710-739)	2/632 (0,3)
All Cancer (140-208)	209/632 (33,1)
Endocrine, metabolic and immunity disorders (240-279)	8/632 (1,3)
Nervous system (320-359)	4/632 (0,6)
Circulatory system (390-459)	310/632 (49,1)
Diseases of respiratory system (460-519)	37/632 (5,8)
Diseases of digestive system (520-579)	16/632 (2,5)
**Maintenance workers**	
Infectious diseases (0-139)	6/336 (2,1)
Diseases of genitourinary system (580-629)	2/336 (0,6)
Accidents and violence (800-999)	26/336 (7,8)
Diseases of the musculoskeletal system and connective tissue (710-739)	1/336 (0,3)
All Cancer (140-208)	91/336 (26,7)
Endocrine, metabolic and immunity disorders (240-279)	5/316 (1,6)
Blood and blood forming organs (280-289)	1/336 (0,3)
Nervous system (320-359)	1/336 (0,3)
Circulatory system (390-459)	160/336 (47,6)
Diseases of respiratory system (460-519)	20/316 (6,3)
Diseases of digestive system (520-579)	23/336 (6,4)
**Office workers**	
Infectious diseases (0-139)	6/189 (3,2)
Diseases of genitourinary system (580-629)	4/189 (2,1)
Accidents and violence (800-999)	6/189 (3,2)
Diseases of the musculoskeletal system and connective tissue (710-739)	1/189 (0,5)
All Cancer (140-208)	38/189 (20,3)
Endocrine, metabolic and immunity disorders (240-279)	4/189 (2,1)
Mental disorders (290-319)	1/189 (0,5)
Nervous system (320-359)	1/189 (0,5)
Circulatory system (390-459)	106/189 (56,0)
Diseases of respiratory system (460-519)	16/189 (8,4)
Diseases of digestive system (520-579)	6/189 (3,2)

**Table 3:** Proportionate Mortality Ratios (PMR), 1969-1998, for Respiratory and bladder Cancer in Cement Workers in Greece

**Site**	**N**	**PMR**	**95% CI**
**Blue-collar maintenance workers**			
*All respiratory cancer*	91	1.91	1.54 to 2.33
*Lung cancer*	85	2.05	1.65 to 2.52
*Larynx*	6	1.32	0.53 to 2.74
*Bladder cancer*	13	1.52	0.85 to 2.55
**Blue-collar maintenance workers**			
*All respiratory cancer*	35	1.7	1.2 to 2.34
*Lung cancer*	31	1.67	1.15 to 2.34
*Larynx cancer*	4	1.71	0.54 to 4.14
*Bladder cancer*	11	1.49	0.78 to 2.59
**Office workers**			
*All Respiratory cancer*	13	0.62	0.34 to 1.03
*Lung cancer*	13	0.7	0.38 to 1.16
*Larynx cancer*	-	n/a	n/a
*Bladder cancer*	2	1.5	0.25 to 4.96

PMR: proportionate mortality ratio; CI: confidence intervals.

Table 3 shows the proportionate mortality of maintenance workers in cement industry. A statistically significant increase in proportionate mortality due to respiratory cancer among maintenance workers has been observed (PMR = 1.7; 95% CI 1.2 to 2.34). In particular, 35 deaths from respiratory cancer have been found (31 deaths from lung cancer and 4 deaths from larynx cancer. The respective PMRs for lung and larynx cancer were 1.67 (95% CI: 1.15 to 2.34) and 1.71 (95% CI 0.54 to 4.14), respectively.

Table 3 shows the proportionate mortality of office workers in cement industry. In this case, 13 deaths from respiratory cancer (all due to lung cancer) were recorded (PMR = 0.62; 95%CI 0.53 to 1.03). Office workers presented lower proportionate mortality from lung cancer than expected (n = 13; PMR = 0.70; 95%CI 0.38 to 1.16). The PMR among the three groups of workers did differ significantly both for all respiratory cancers and for lung cancer, respectively (p = 0.001). The PMR for urinary bladder cancer was similar among the three groups of cement workers.

Table 4 presents the proportionate mortality (cement production workers only) from all cancer, ischemic heart disease, cerebrovascular disease and pneumonia.

**Table 4:** Proportionate Mortality Ratios (PMR), 1969-1998, for all cancer, ischemic heart disease, cerebrovascular diseases and pneumonia in Cement Production Workers in Greece

**Site**	**N**	**PMR**	**95% CI**
**Cement production workers**			
*All cancer (140-239)*	209	1.3	1.13 to 1.49
*Ischemic heart disease (410-414)*	111	1.37	1.12 to 1.64
*Cerebrovascular diseases(430-438)*	91	1.11	0.89 to 1.35
*Pneumonia (480-486)*	14	1.75	0.99 to 2.86

## Discussion

The evidence regarding lung cancer and occupational exposure to cement is controversial. However, there is some evidence that it is biologically plausible that cement dust could have the potential to cause cancers at sites of contact. As a highly alkaline substance, cement can cause irritation at sites of contact, such as the mouth, throat, and lungs. Persistent chronic irritation will cause repeated cycles of cell death, cell proliferation and other inflammatory responses. It is recognized that this process can be a step on the pathway to cancer [2]. The present study suggests that cement workers (cement production and maintenance) present significantly elevated all respiratory and lung cancer mortality. On the contrary our study failed to suggest a significant increase of proportionate mortality from laryngeal cancer; however both PMRs were elevated based on small numbers. There is conflicting evidence on the association between occupation exposure to cement dust and risk of respiratory (lung and laryngeal) cancer. Jakobson et al, in a Swedish retrospective cohort study, did not find an increased mortality from respiratory cancer [8]. Dab and colleagues, in a French cohort study with a follow up period of 15 years, also failed to demonstrate an elevated mortality from respiratory cancer among workers of a cement industrial plant [9]. Giordano et al, in an Italian cohort study, found a significantly elevated mortality for respiratory cancer, exclusively in a group of cement workers with previous exposure to asbestos [10]. On the other hand, Smailyte and coworkers in a cohort study among workers of the Lithuanian cement industry plan, found an excess in Standardised Mortality Ratio (SMR) for lung cancer [7]. Rafnsson et al, in an Icelandic cohort study, found an increased risk of lung cancer among masons [6]. Moreover, a case-control study commissioned by the International Agency Research Cancer (IARC) in Central and Eastern Europe, found that occupational exposure to cement dust was associated with an increased risk of lung cancer (Odds Ratio = 3.62; 95%CI 1.11 to 12)[12]. Compared to the previous studies, the present study has the considerable advantage to rely on data from the insurance scheme of cement workers. Therefore, our results may have the potential for a satisfactory level of generalizability.

Our study presents several limitations which need to be discussed. A first limitation is related to the PMR analysis employed in this study. Proportionate mortality analysis is used when the total population at risk is not known and only death information on the cohort under study is available. Consequently, proportionate mortality analysis does not provide a true estimate of the risk. Another limitation of our study is related to the exposure assessment. Since historical exposure data (industrial hygiene measurements) were not available, the job title was used as surrogate indicator of exposure to cement dust. In fact, despite of the presence of detailed information on employment of the workers in the cement industry, we cannot rule out that information bias in terms of exposure misclassification may have occurred. Another possible limitation of the present study is related to the confounding role of smoking. Our study lacks longitudinal information on smoking habit of the workers in the cement industry. This is also the case with almost all studies reporting mortality data from cement workers. However, we believe that is unlikely that our finding of the significantly elevated proportionate mortality from lung cancer could be due to the confounding effect of smoking. If that was the case, then the smoking habit would largely differ between production, maintenance and office workers. However, there are data suggesting that blue and white collar workers in Greece have comparable rates of smoking [13]. Therefore we believe that there is no obvious reason that smoking habit should differ substantially between these categories of blue collar workers. Moreover, Axelson suggested that confounding from smoking would rarely explain risk ratios of more than about 1.5 for lung cancer [14]. Based on the aforementioned data, it is likely that the confounding effect of smoking could be considered as minimal in the present study. This argument is supported by our finding regarding the comparable levels of bladder cancer proportionate mortality across the three categories of cement workers. Regarding the possible confounding effect of asbestos exposure the fact that no death from mesothelioma was found among cement workers suggests that asbestos does not represent a considerable confounding factor in the present study.

Moreover, an additional limitation of the proportionate mortality methodology is that the magnitude of each cause of death is dependent upon the magnitude of the proportionate mortality for other causes of death. If the PMR for the common cause of death is high, the PMRs for the other causes are artificially deflated, and vice versa [15]. It is well known that in a working population the risk for diseases of the circulatory system is lower due to the ‘’healthy worker effect”, and therefore, in a proportionate mortality study the other causes could be artificially increased. However, this is not the case in the present study where diseases of the circulatory system were the prevalent cause of death. Moreover, the PMRs for two major causes of death ischemic heart disease and cerebro-vascular diseases (cement production workers only) were 1.37 and 1.11, respectively. Thus, it seems unlikely that the elevated PMR for lung cancer could be attributed to an under-representation of major causes of death like CHD, and CVD.

Last, an interesting finding of our study which deserves further future investigation was the elevated proportionate mortality from pneumonia among cement production workers. There is some evidence that exposure to inorganic dust increases the mortality from infectious pneumonia. It has been suggested that this biological effect could be mediated through induced airways inflammation [16].

## Conclusion

In conclusion, the present study, based on data from a large registry from the insurance scheme of cement workers in Greece, suggests that cement production workers present increased respiratory and lung cancer mortality, and this finding probably cannot be explained by the confounding effect of smoking alone. Further research including use of prospective cohort studies is needed in order to establish a causal association between occupational exposure to cement and risk of lung cancer.

## Competing interests

The authors declare that they have no competing interests.

## Authors’ contributions

GR participated in study design, data analysis and drafted the manuscript. SD participated in study design and interpretation of data. KK revised the manuscript for important intellectual content. VM participated in study design, interpretation of data and revision of the manuscript for important intellectual content. CH participated in study design, supervised data analysis, preparation and revision of the manuscript for important intellectual content. All authors have read and approved the manuscript.

## Pre-publication history

The pre-publication history for this paper can be accessed here:

http://www.biomedcentral.com/1471-2466/12/30/prepub
